# Longitudinal assessment of competency development at The Ohio State University using the competency-based veterinary education (CBVE) model

**DOI:** 10.3389/fvets.2022.1019305

**Published:** 2022-10-24

**Authors:** Emma K. Read, Connor Maxey, Kent G. Hecker

**Affiliations:** ^1^College of Veterinary Medicine, The Ohio State University, Columbus, OH, United States; ^2^Faculty of Veterinary Medicine, University of Calgary, Calgary, AB, Canada; ^3^International Council for Veterinary Assessment, Bismarck, ND, United States

**Keywords:** longitudinal assessment, ITER, in-training evaluation report, EPA, entrustable professional activity, entrustment, milestones, CBVE

## Abstract

With the development of the American Association of Veterinary Medical Colleges' Competency-Based Veterinary Education (CBVE) model, veterinary schools are reorganizing curricula and assessment guidelines, especially within the clinical rotation training elements. Specifically, programs are utilizing both competencies and entrustable professional activities (EPAs) as opportunities for gathering information about student development within and across clinical rotations. However, what evidence exists that use of the central tenets of the CBVE model (competency framework, milestones and EPAs) improves our assessment practices and captures reliable and valid data to track competency development of students as they progress through their clinical year? Here, we report on validity evidence to support the use of scores from in-training evaluation report forms (ITERs) and workplace-based assessments of EPAs to evaluate competency progression within and across domains described in the CBVE, during the final year clinical training period of The Ohio State University's College of Veterinary Medicine (OSU-CVM) program. The ITER, used at the conclusion of each rotation, was modified to include the CBVE competencies that were assessed by identifying the stage of student development on a series of descriptive milestones (from pre-novice to competent). Workplace based assessments containing entrustment scales were used to assess EPAs from the CBVE model within each clinical rotation. Competency progression and entrustment scores were evaluated on each of the 31 rotations offered and high-stakes decisions regarding student performance were determined by a collective review of all the ITERs and EPAs recorded for each learner across each semester and the entire year. Results from the class of 2021, collected on approximately 190 students from 31 rotations, are reported with more than 55 299 total competency assessments combined with milestone placement and 2799 complete EPAs. Approximately 10% of the class was identified for remediation and received additional coaching support. Data collected longitudinally through the ITER on milestones provides initial validity evidence to support using the scores in higher stakes contexts such as identifying students for remediation and for determining whether students have met the necessary requirements to successfully complete the program. Data collected on entrustment scores did not, however, support such decision making. Implications are discussed.

## Introduction

Veterinary medicine recently began adoption of competency-based education (CBE) and this has been formalized by introduction of three components of the competency-based veterinary education (CBVE) model: CBVE Competency Framework, Entrustable Professional Activities and Milestones (1–4). CBE is widely employed across health sciences education with emphasis on a learner-centered approach and recurring assessments (5). CBE has moved beyond evaluation of learning for the knowledge domain and is inclusive of psychomotor skills, attitudes and values (5). Since single assessments do not adequately capture all these dimensions at once, longitudinal assessment using multiple assessment methods has become a necessity to gather the complete picture of learning or competency across the domains (6).

Longitudinal assessment utilizes a data continuum to provide information about an individual's learning and includes a mixture of low-stakes opportunities and high-stakes decisions (7). What is presented in this study are the preliminary building blocks for a programmatic approach (8) where assessment data are collected longitudinally and periodically reviewed by an oversight clinical educators' committee that renders high-stakes progress decisions regarding learners (5). Here, we report on the use of two assessment methods that incorporate scales which have been reported to result in more reliable and valid scores over time (9). The intent of using these data are to provide students and stakeholders with feedback regarding competency progression through clinical rotations. Before fully committing to an oversight committee, sometimes referred to as a competence committee (5) and a more detailed programmatic assessment approach, here we first wanted to ensure that data can be collected according to a competency profile and entrustable activities, where data regarding milestones and entrustment scales result in reliable scores to assist with identifying students earlier for remediation and demonstrate clear evidence of learner growth over time.

So why the push to adopt CBE or CBVE at all? Increasingly educators are finding value in the demonstration of “assessment as learning” with frequent specific feedback providing more meaning to learners and helping to better guide their development (10).

Convincing evidence for student learning in all domains of competence is being increasingly demanded by educators, accrediting bodies, and stakeholders alike (10). In medical education, a recent study compared competencies measured using Likert type scales to those using entrustment scales (11) and national studies are being conducted to compare milestone ratings in resident training programs with national licensing examination scoring (12). Veterinary specific examples are more limited to date however there has been demonstration of strong validity evidence for a programmatic assessment approach (13). This veterinary study used VetPro (14) as the competency framework with the item scales on the workplace-based assessment methods ranging from 1 (novice) to 5 (expert).

EPAs are described as the daily activities of a practicing veterinarian that can be used as opportunities to observe performance and offer feedback using workplace-based assessment methods (15). To clarify further, competencies describe the veterinarian, while EPAs describe what the veterinarian does (16). Pilot work at the University of Calgary identified an entrustment scale that proved useful with assessing students in general practice veterinary settings in a distributed veterinary teaching hospital and this was used as the basis of establishing a scale for use at OSU-CVM which featured 5 anchor points: Don't trust to perform any aspect of the task, Trust but needs lots of help, Trust but needs some help, Trust but needs on-demand guidance, Trust to perform on own (17). OSU-CVM used EPAs from the CBVE model with EPA 1 separated into 3 individual EPAs, so 10 EPAs were made available for learners to select from.

Here, we report both the use of the CBVE milestone scale within an In-training Evaluation Report (ITER), and the use of scores from entrustment scales for evaluation of CBVE EPA performances. The work here with its underlining assumptions builds our validity argument (18, 19) that scores from these methods will provide reliable and valid evidence to support progression and remediation decisions for final year veterinary students. To affirm this, scores would need to be assessed for their reliability (consistency of scores within and across method overtime) and their validity (changes in scores over time to demonstrate learning progression), and scores would also need to demonstrate a consistent positive correlation between milestone scores and entrustment scores. Such findings could then be used to produce preliminary evidence for scoring and generalization within Kane's argument-based validity framework (18, 19).

With this as background, the purpose of this research is to report on the validity evidence of scores from both in the moment assessments (of EPAs) and clinical rotation milestone scores on competency development, within and across domains, within and across time, over the clinical component of a veterinary program.

## Methods

### Context

This study was provided exemption from IRB review by The Ohio State University Office of Responsible Research Practices. OSU-CVM has a final year program that includes 31 possible rotations offered across five hospitals in the Veterinary Health System. Each final year student is enrolled in three identical courses that are graded “S” (satisfactory) or “U” (unsatisfactory). Each course spans a single semester and the final year program is comprised of three semesters, that include 8 rotations each. The rotations selected for each student will vary according to the career area of emphasis they select: small animal, farm animal, mixed animal, equine, spectrum of care, and individualized (research, public health). Each student has a total of 24 rotations chosen from a total of 31 available. Beginning with the class of 2021, changes were made to the final year assessments to move from a single summative judgement at the conclusion of each rotation to a suite of workplace-based assessment methods that emphasize student development over time. OSU-CVM introduced a new ITER based on milestone progression, as well as EPAs and workplace-based assessment with corresponding entrustment scales for timely direct observation with formative feedback. The college also required student reflection on their longitudinal competency development across rotations at the end of each semester and created opportunities for more faculty coaching input (not reported here). The 2020–21 final year program was 25 blocks in length (including 2 weeks vacation) with the first 5 blocks being conducted on-line because of the global COVID-19 pandemic. In the first 5 blocks all students were required to take an orientation, followed by rotations in diagnostic imaging, applied pathology, clinical pathology, and preventive medicine. The remaining 20 in-person 2-week long rotations were offered across the veterinary health system. Data are shown from Block 6 onwards.

### ITER based on milestone progression within competencies

A modified ITER was created with adoption of the CBVE's 32 competencies and associated four milestones (novice, advanced beginner, competent and proficient). A fifth milestone (pre-novice) was added following consultation of OSU-CVM faculty and was defined for all competencies as “not yet meeting novice level”. This was introduced because faculty members had expressed concerns that some students may arrive into the clinical training portion of the program without yet being able to meet the novice milestone in all competencies. Faculty did not wish to cause students to lose confidence in their training to date should this occur, and they also wanted to encourage learners to keep progressing toward higher level milestones. Brief video training materials were created to orient learners and raters to the assessment process and to encourage standardized use of the tools across rotations. As part of the development, each of the rotation coordinators were asked to identify the competencies from the CBVE framework that their respective rotation could assess in each learner that attended their rotation. The number of observations of each competency are reported in the results (Table 1). The premise at the time was that the sum of the assessment pieces from each rotation would provide a complete picture of overall development as the year progressed and as the learner navigated the 20 clinical rotation blocks that made up the remaining final year program.

**Table 1:** Summary of the observational data: number of observations made per competency, number of students assessed per competency, number of raters providing feedback, mean number of assessments provided per student for each competency, and mean number of ratings provided per rater for each competency.

**Competency**	**No. observations**	**No. students**	**No. raters**	**Mean no. assessments/student**	**Mean no. ratings/rater**
1.1	5075	198	140	26	36
1.2	5075	131	30	5	20
1.3	5042	131	30	5	20
1.4	1960	131	30	5	20
1.5	1899	131	30	5	20
1.6	2140	131	30	5	20
1.7	2224	131	30	5	20
2.1	2584	81	30	5	66
2.2	526	81	4	3	66
3.1	206	12	4	3	9
3.2	37	12	4	3	9
3.3	647	12	4	3	9
4.1	299	68	8	3	26
4.2	271	68	8	3	26
5.1	5045	172	40	6	24
5.2	1848	172	40	6	24
5.3	2287	172	40	6	24
6.1	510	75	5	1	16
6.2	2989	75	5	1	16
6.3	793	75	5	1	16
6.4	306	75	5	1	16
7.1	226	100	12	2	19
7.2	5090	198	140	26	36
7.3	2632	198	73	13	36
7.4	2100	197	82	11	26
7.5	174	114	9	2	19
8.1	206	68	8	3	26
8.2	647	167	11	4	59
8.3	300	129	10	2	30
9.1	311	176	27	3	19
9.2	1580	196	51	8	31
9.3	70	60	2	1	35

**Figure 1:**
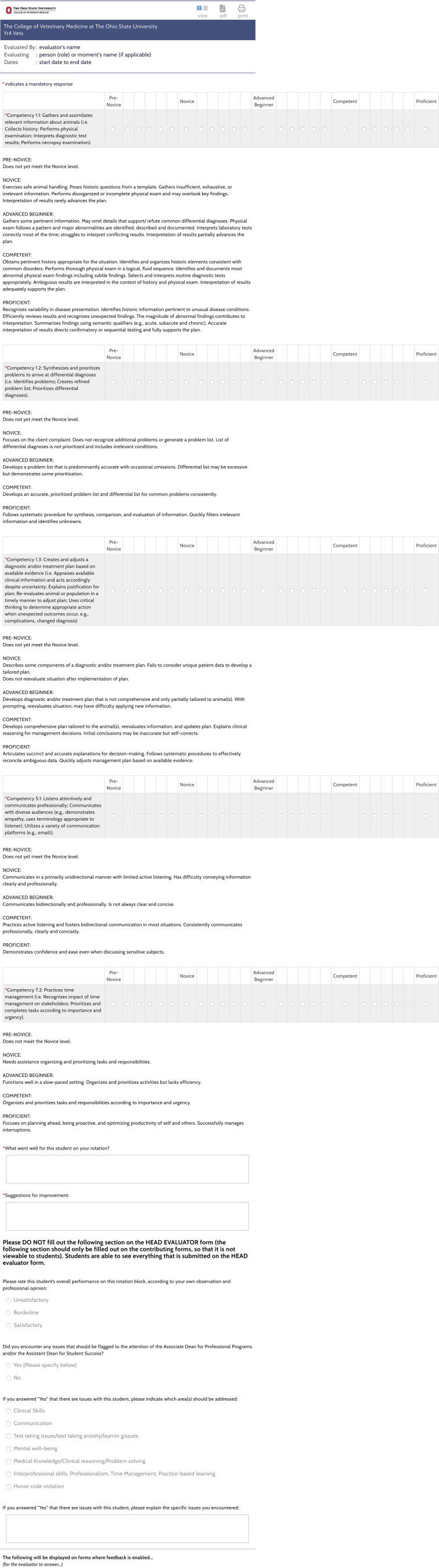
The Ohio State University College of Veterinary Medicine final year rotation ITER (in-training evaluation report) form showing the use of a “bubble bar” for continuous scoring across milestone anchors.

Any clinician or preceptor within a rotation who worked with a student was able to complete an ITER form and for each rotation all the forms were averaged together to create a ‘total ITER form’ that assigned each student their mean final milestone rating for each competency assessed. Placement of student performance on the CBVE milestone anchors involved the use of a ‘bubble bar’ visual analog scale that incorporated a 25-point scale with equal intervals between milestones. A description of each milestone anchor was provided for each different competency as per the CBVE model (4). This provided the rater flexibility in reporting their observation of student's ability within competency. See Figure 1— The ITER form. Clinicians were asked to provide qualitative comments specific to what went well and what areas needed to be addressed by the learner for further development in the competency area. Clinicians also had the opportunity to “flag” to the Associate Dean for Professional Programs if the student's performance prompted concerns as it related to: clinical skills, communications, mental wellbeing, medical knowledge, clinical reasoning and critical thinking, interprofessional skills, professionalism, time management, and honor code violation. Free text comments were also encouraged to confidentially report the clinicians' observations to the Associate Dean so that a remediation program could be developed to directly address the deficiencies if required.

The “total ITER form” and qualitative comments was ultimately reviewed by each rotation leader and a “global total ITER” score of “S”—satisfactory, “B”—borderline, or “U”—unsatisfactory was recorded. Any discrepancies between milestone scoring, qualitative comments or “flags” were checked by the Associate Dean in consultation with the rotation leader to ensure appropriate interpretation.

A final rotation grade of “S” (satisfactory) or “U” (unsatisfactory) for the rotation was assigned later following use of a borderline regression analysis that plotted mean overall score (from across all domains and competencies scored on the 25-point scale) vs. the “global total ITER” score (S, B or U). The final rotation grade was therefore not determined by the observing clinical faculty but was instead based on review of the borderline regression analysis results, the qualitative comments, and EPAs. The “global total ITER” score assigned by rotation leaders assisted in the analysis but was not the final score for the rotation.

### Data analyses

Data from the 2020–21 academic year were analyzed using SPSS v.28 (IBM, Chicago, IL, USA). Generalizability theory, where student was crossed with competency nested within clinical rotation was used to calculate reliability coefficients for the ITER. To assess progression of performance across clinical rotations, composite learning curves were built to determine if milestone scores were different across students, if scores changed over time, and if we can differentiate between competencies. Given that repeated milestone scores (level 1) were nested within CBVE competencies (level 2) nested within student (level 3), a multilevel random coefficient model was used to model variance components for each of these levels, similar to Bok et al. (13). Within student (level 3), clinical rotation block was included to assess whether students begin at the same level (intercept variance) and progressed at the same rate (slope variance) over time. Finally, as reported by Bok et al. (13), these data may change due to modeling linear and non-linear time, therefore data were further analyzed with linear, quadratic and cubic functions and significant changes were assessed using the−2log likelihood chi-square analyses.

## Results

Every OSU-CVM rotation (except for preventive medicine) identified the same 5 competencies in common −1.1, 1.2, 1.3, 5.1, and 7.2, however not all the CBVE competencies were assessed on every rotation. All of the competencies were assessed for each student multiple times over the year as they progressed through their rotations. See Table 1 for further details on the number of observations of each competency. See Table 1 and three for Generalizability and multilevel model analyses report for the 5 competencies mentioned above.

During 2020-21, across the final year clinical program there were 55,299 total competency assessments using ITERs and 2,799 complete EPAs collected on 190 students.

**Figure 2:**
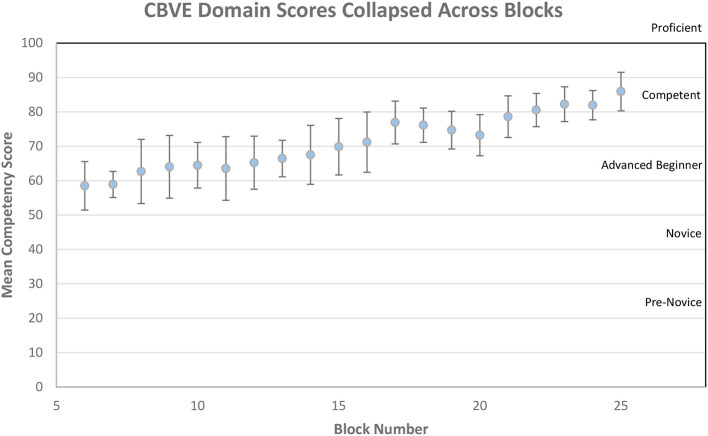
Mean competency scores over time for entire cohort collapsed across all CBVE domains.

**Figure 3:**
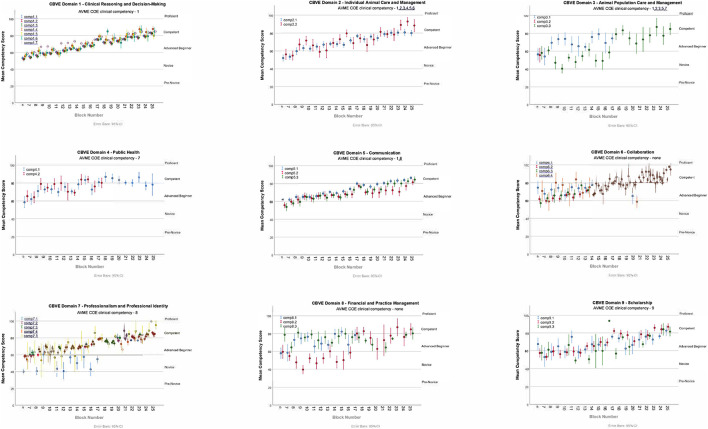
Mean competency scores over time for entire cohort for each individual CBVE domain.

Figure 2 shows the visual representation of the average competency growth for the whole cohort across all of the domains and competencies (collapsed together) for the VME IV clinical program in 2020–21. Figure 3 shows the visual representation of the average competency growth for the whole cohort across each of the 9 CBVE domains and 32 competencies for the final year clinical program in 2020–21 according to data collected from these ITERs.

Table 1 shows the observational data collected from the ITERs: number of observations per competency, number of students assessed per competency, number of raters contributing to each competency assessment, as well as the mean number of assessments per student for a particular competency, and the mean number of ratings provided by each clinician for each domain.

Table 2 reports the reliability (G-coef) as 0.85. This suggests milestone ratings of students were consistent across competencies and blocks. Greater variance was accounted for student within block (43.16%; indicating scores discriminate high and low performing students consistently) compared to competency within block (1.67%; little discrimination by competency score) and by week (17.50%; scores change over weeks).

**Table 2:** Generalizability Analysis for rotation block (*n* = 24), student nested within block (*n* = 191) and competency nested within block (*n* = 5 competencies).

**Source**	**Variance**	**%**
Block	77.98	17.50
Student within block	192.34	43.16
Competency within block	7.46	1.67
Error	167.83	37.66
Total	445.611	
G-coeff	0.85	

The random coefficients model (Table 3) nests repeated scores within competency domain within student with time fixed (Model 1) and time modeled as a linear function (Model 2) as suggested in Bok et al. (13). In the first model (Model 1) students' intercepts account for the greatest amount of variance (79.66%) indicating students start at different milestone ratings. The slope indicates that rate of student learning differs negligibly between student (student progress at the same milestone rating over time). The covariance term in level 3 of the model was negative indicating higher initial milestone scores have a less positive change over time. Interestingly there is no variance between competency, suggesting milestone ratings among competencies vary little within rotation. Finally, the residual includes the variance due to repeated measures nested within competency and student (20.06%).

**Table 3:** Multilevel random coefficients models, where repeated measures are nested within competency and students.

	**Model 1 (SE)**	**% of variance**	**Model 2 (SE)**	**% of Variance**
**Fixed effects**				
Intercept	70.84 (0.51)*		45.66 (0.99)*	
Week			1.53 (0.05)*	
Week*week				
Week*week*week				
**Random effects**				
Level 1: Repeated measures (residual)	200.00(2.34)*	20.06	200.00 (2.37)*	54.79
Level 2: Competency domain (intercept)	0	0	0	
Level 3: Student (intercept)	794.05 (102.22)*	79.66	164.66(20.33)*	45.11
Level 3: Student (covariance)	−45.51 (5.18)*		−6.57 (0.97)*	
Level 3: Student (slope)	2.77 (0.29)*	0.28	0.38(0.05)*	0.001

Model 2 models a sigmoidal learning curve on the repeated measures.

Model 2 assumes changes in milestone ratings are constant (linear) over time. There is an improved model fit (χ2(1) = 1,20,065.83–1,19,752.72 = 331.10, *p* < 0.01). The value of 1.53 is the expected change in student milestone ratings over one block. Given that there are 19 total blocks (clinical rotations) this represents an average student increase of 29.07 indicating a significant change in milestone rating. There is a difference in student variance between Model 1 and Model 2 due to change over time (794.05–164.66 = 629.39). The general trends that were present in Model 1 are reflected in Model 2, the majority of the variance was due to different student intercepts (different milestone ratings; 45.03%), the rate of change does not change between students (slope variance in minimal) and there is again no variance between milestone competency assessment. When we modeled time as a quadratic function (one inflection point) there were no differences in the amount of variance that the model accounts for (χ2(1) = 1,19,752.72–1,19,755.19 =-2.44, *p* = n.s.), therefore we did not pursue further data analysis modeling time as non-linear.

**Figure 4:**
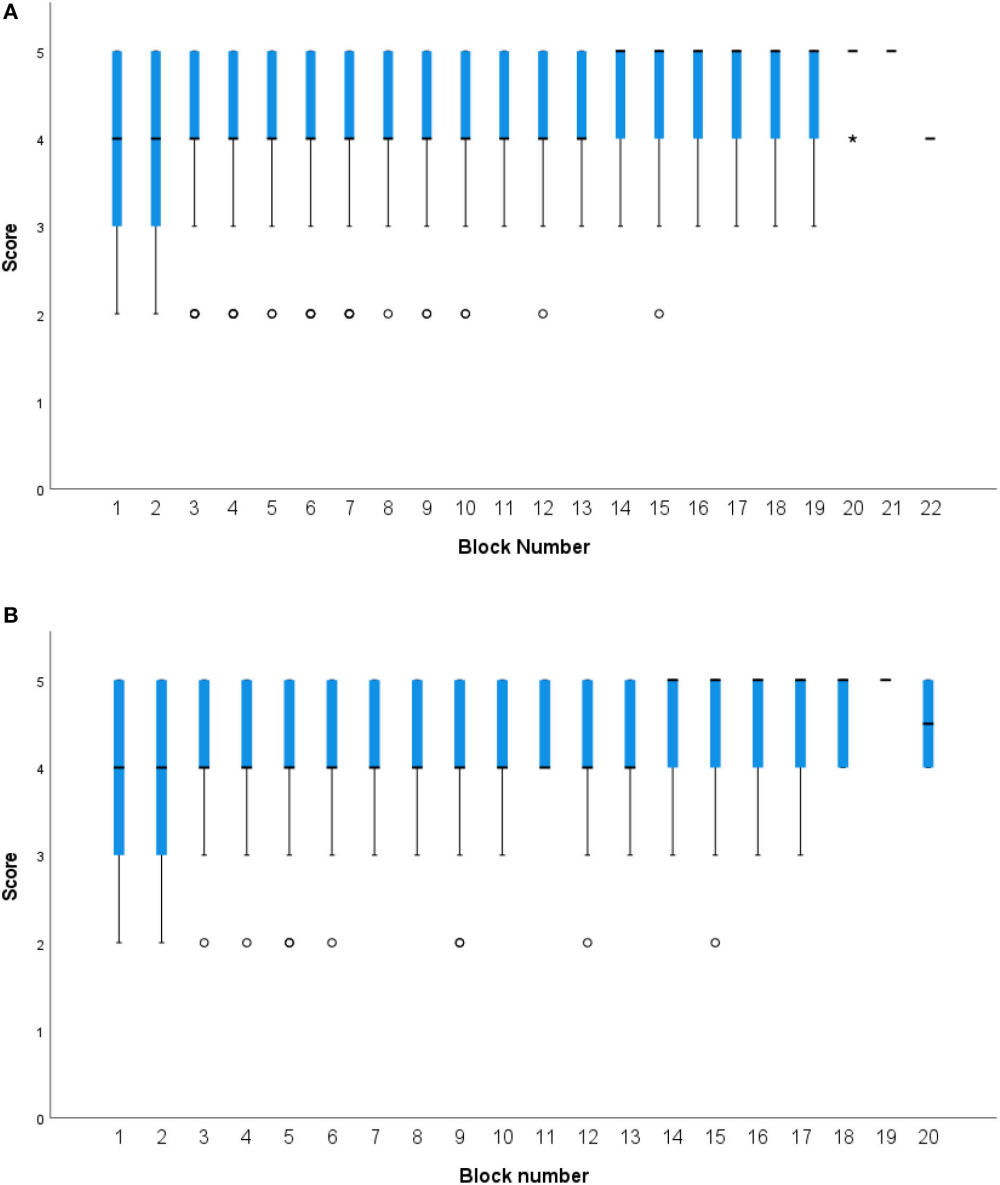
Box plots presenting categorical entrustment scores on EPAs across clinical rotation blocks. **(A)** Combined entrustment scores across all EPAs by rotation block. **(B)** Entrustment scores for the “Performs a Physical Exam” EPA, the EPA most assessed within and across rotation blocks.

Table 4 shows the number of students choosing to complete various EPAs. Using entrustment scales as the assessment tool, performance within the various EPAs was shown not to vary over time. Figure 4 shows entrustment scores for EPAs across rotation blocks and specifically, for the most assessed EPA “Performs a Physical Exam.”

**Table 4:** Number of students selecting to complete each EPA.

**EPAs**	**Count**	**%**
1a. Gather a history	424	11%
1b. Perform an exam	1,368	35%
1c. Create a prioritized differential diagnosis list	265	7%
2. Develop a diagnostic plan and interpret results	493	13%
3. Develop and implement a management/treatment plan	503	13%
4. Recognize a patient requiring urgent or emergent care and initiate evaluation and management	72	2%
5. Formulate relevant questions and retrieve evidence to advance care	51	1%
6. Perform a common surgical procedure on a stable patient, including pre-operative and post-operative management	346	9%
7. Perform general anesthesia and recovery of a stable patient including monitoring and support	267	7%
8. Formulate recommendations for preventive healthcare	67	2%

## Discussion

The main findings of this research are: Milestone scores from ITERs demonstrate adequate reliability (G coef = 0.85). The multilevel model indicates that students begin the final year clinical program ranked at different milestone markers, their performance increases at relatively the same rate as they progress through their clinical rotations, yet faculty assessment between core competencies was not distinguishable. Interestingly there was little variability in entrustment scale scores for the EPAs assessed across rotations.

The unique contributions to veterinary medical education assessment here are two-fold. First, there is an uptake and use of items such as milestones where preceptors/faculty appear to assess student performance consistently within and across rotations and use the scores for student feedback and development. Also there is the implementation and use of EPAs and entrustment scores. However, there was little to no differentiation in entrustment scores of students. There are a number of potential factors why faculty and students may not have used the EPA framework and entrustment scales as intended which include: assessor understanding of the EPAs and their use, assessor understanding of the entrustment scales and their use, and the broad range of assessors completing entrustment scales (including faculty, technical staff, residents and interns). The latter population is different to those completing ITERs (faculty and residents only) who represent a group with more education and assessment experience collectively and by comparison. In addition to differences in how the tools are completed there may be differences in how they are being interpreted by students and used by administration. Entrustment scales were used here as low stakes assessment tools which may have diminished their perceived value for assessors and resulted in less discriminating selection between anchor points with a focus on being just good enough. In other veterinary programs entrustment scale tools and EPAs have been reported for use in a high-stakes capacity (13, 20).

The second unique contribution of this work is demonstration of learner progression over time, supported by our analysis. These findings advance use of the CBVE model components for effective scoring of learners in veterinary medicine.

### ITERs: Competencies and milestones

Despite individual students who occasionally rated as pre-novice, there was no rating of the entire cohort below novice for any individual competency or domain, and the cohort achieved competent for all competencies by the end of the training period (Figures 2, 3). Figure 3, showing milestone by domain, could be reflective of OSU's pre-clinical training and how this contributes to their entry-level performance upon arrival into the clinical workplace (21). For example, in domain 1 (individual animal care and management) the initial assessments (as represented near the y-intercept) falls at the novice milestone for performance, yet in domain 5 (communication) the initial assessments (as represented near the y-intercept) falls at the advanced beginner milestone. These milestone ratings may reflect the robust communication training program that has been in place for almost a decade, while the clinical skills program is a more recent addition to the program. These examples highlight that while the multi-level model did not show variance due to competency there are some interesting findings when the Domain scores are considered. Domains 3 (animal population care and management) and 8 (financial and practice management) were more difficult to interpret due to fewer specific data points and because they demonstrated less readily identifiable learning curves compared to the other domains. These particular domains were less frequently identified by rotation leaders as ones that could be reliably assessed in every student on every rotation resulting in fewer data collected. This reinforces that thorough assessment of student progression in all areas requires evaluation of multiple competencies across the entire program and should likely span more than just the clinical training period (6). Fortunately, domain 3 (animal population care and management) is heavily assessed in pre-clinical core courses and competency 8.1 (economic factors in personal and business decision making) is a significant component of the professional development courses.

The ITERs allowed identification of approximately 10% of students per semester that could benefit from remediation. Students participating in remediation activities are required to have formal reporting from supervising instructors and they have all shown improvement in order to progress. Any student required to repeat a rotation is assessed using the same ITER form and to date, all students have shown improvement in their scoring or qualitative comments on the repeated experience. Repeat rotations are typically undertaken with different rotation instructors wherever possible to avoid conflict of interest with grading.

A recent paper described that validity evidence for an assessment system can be supported with demonstration that growth in performance over time follows a theoretically predictable pattern (such as learning curves) (9). Figure 3 shows such patterns for all domains of competence that were assessed in learners at OSU-CVM using the CBVE.

### Entrustment scales and EPAs

Studies in medicine have shown the importance of using methods that focus on both the granular and the holistic in programs of assessment, with emphasis being placed on EPAs to provide narrative value (22). EPAs were introduced at OSU-CVM to create opportunities to provide direct observation of performance and facilitate provision of formative feedback using an entrustment scale tool. All students must complete one EPA per rotation block as a program requirement and the student initiates the process by selecting an EPA and asking to be observed performing it. The most commonly selected EPA was 1b “perform a physical exam” (35%), followed by “develop a diagnostic plan and interpret results” (13%), “develop and implement a management/treatment plan” (13%), and “gather a history” (11%). It is likely that these were the most commonly selected EPAs because students already had some comfort and familiarity with these activities and thought that they may have greater success in performance “under pressure.” It may also be that these activities are simply those that a student performs multiple times per day repetitively on each block and it was instead a matter of convenience.

The least commonly selected EPAs were recognizing a patient needing urgent or emergent care (2%), formulate relevant questions and retrieve evidence to advance care (1%), or formulate recommendations for preventive healthcare (2%). The avoidance of selecting the EPA for urgent or emergent care is likely because emergency rotations tend to be fast paced, high-stress learning environments leaving little time to ask questions (23). This makes it difficult to ask a clinical assessor to interrupt an urgent situation to allow a student to take on the lead role. Alternatively, it may also be an avoidance on the part of the student to avoid making higher stakes decisions when time is tight and the situation is urgent. Further investigation is required to determine why student preferences occur as they do. Recall, OSU students are allowed to freely select any EPA they wish to be evaluated on for their single mandated EPA per block. A recent discussion amongst the final year teachers affirmed that the most frequently selected EPAs tend to be amongst some of the most important to a new graduate and therefore rotation leaders were content with students having multiple measures of assessment on the same activities repeatedly and consider this valuable repeated practice over time.

Figure 4 shows combined mean entrustment scores assigned for EPAs across all rotations and the entrustment scores assigned for the “performs a physical exam” EPA. The box plots show that there is relatively little variation in scoring in either the overall cohort or individual student consideration. As mentioned above, the reasons for this lack of variability could be the focus of further research and will warrant further consideration of how best to incorporate entrustment scales into veterinary medical assessment. Other health profession fields report that frontline faculty are struggling with entrustment-anchored scales, and while that may not be the case in veterinary medicine, it's clear that we have much to learn about their implementation (24).

## Conclusion

The development of ITERs based on milestones and competences and Entrustment Scale forms at OSU-CVM was modeled on the CBVE model and a previously validated entrustment scale used at the University of Calgary (17). There were changes over time from novice to competent for each domain and competency on ITER scores. Analyses provide foundational support for the reliability of the scores and some supportive validity evidence on which to make important progress decisions. However, entrustment scores provided little differentiation between and within students, on EPAs, over the clinical rotation period. To incorporate and use scores from both methods (in conjunction with other clinical rotation assessment methods) to make competency decisions in a programmatic assessment framework requires further training of all stakeholders in order to maximize their intended use. Gathering of multiple data points per learner over time, using various assessment tools, is key to a more holistic assessment of student ability and is consistent with providing the foundation for more evidence-based practice of programmatic assessment within veterinary medicine.

## Data availability statement

The raw data supporting the conclusions of this article will be made available by the authors, without undue reservation.

## Ethics statement

The studies involving human participants were reviewed and approved by The Ohio State University Office of Responsible Research Practices. Written informed consent for participation was not required for this study in accordance with the national legislation and the institutional requirements.

## Author contributions

ER conceived, led the design of the assessments, and conducted the study. CM organized the database and performed the statistical analysis. KH advised on the assessment design and statistical analysis. All authors contributed to the manuscript revisions and approved the final version.

## Conflict of interest

Author KH is the Chief Assessment Officer for the International Council for Veterinary Assessment. The remaining authors declare that the research was conducted in the absence of any commercial or financial relationships that could be construed as a potential conflict of interest.

## Publisher's note

All claims expressed in this article are solely those of the authors and do not necessarily represent those of their affiliated organizations, or those of the publisher, the editors and the reviewers. Any product that may be evaluated in this article, or claim that may be made by its manufacturer, is not guaranteed or endorsed by the publisher.
